# Upper Extremity Deep Vein Thrombosis in the Setting of Ductal Carcinoma In Situ: A Case Report

**DOI:** 10.7759/cureus.61805

**Published:** 2024-06-06

**Authors:** Ahmed Elashmawy, Linsey Gold

**Affiliations:** 1 Surgery, Wayne State University School of Medicine, Detroit, USA; 2 Surgery, Oakland University William Beaumont School of Medicine, Rochester, USA

**Keywords:** screening mammogram, tumor imaging, upper extremity thrombosis, breast cancer management, ductal carcinoma in situ (dcis)

## Abstract

Deep vein thrombosis (DVT) is a type of venous thromboembolism that usually involves a clot formation in the deep veins of the lower extremities. Its formation is linked to Virchow’s Triad which factors in venous stasis, endothelial damage, and hypercoagulability. Venous stasis is the primary factor contributing to the development of DVT and it refers to varicosity, external pressure placed on the extremity, or immobilization due to bed rest or long flights. Clinical presentation of DVT depends on the extent and location of the thrombus with common signs including localized swelling, pain, warmth, and edema. The Wells criteria are typically applied to assess the likelihood of thrombus formation alongside D-dimer assay, ultrasound, or CT imaging. As previously mentioned, these mostly occur in the lower extremities. However, upper extremity DVT has been noted and has been linked to inherited issues with coagulation and autoimmune disorders. This report will discuss a case of left-arm DVT in a patient who underwent bilateral mastectomy with sentinel node biopsy for a diagnosis of ductal carcinoma in situ in the left breast.

## Introduction

Deep vein thrombosis (DVT) tends to occur in areas with decreased or mechanically altered blood flow such as the pockets adjacent to valves in the deep veins of the leg [1]. It is classified by the position of anatomical occurrence. Proximal DVT involves the femoral vein, profunda femoris vein, or popliteal vein, while distal DVT typically involves veins below the knee joint or beyond the calf vein trifurcation [2]. Surgical risk factors associated with DVT development include surgery under general anesthesia and cesarean delivery. Estrogen (ER)-related factors associated with DVT include pregnancy and the use of oral contraceptives or hormone replacement therapy. Patient factors that are correlated with the development of DVT include obesity, smoking, and age > 60 years. Certain metabolic states such as cancer, autoimmune disorders, and genetic mutations, among which are factor V Leiden, antithrombin III deficiency, protein C deficiency, and protein S deficiency, can also contribute to the development of DVT [3,4]. A few of the factors mentioned above will be discussed in the following report detailing a patient who presented with DVT in the upper extremity in the setting of malignancy. 

## Case presentation

A 51-year-old female with a past medical history of skin cancer, hypothyroidism, depression, and anxiety was found to have ductal carcinoma in situ (DCIS) of the left breast on a routine screening mammogram. Gynecological history revealed that the patient is premenopausal, experienced menarche at the age of 10, and denies ever having breastfed. Genetic testing for hereditary breast cancer was negative for BRCA 1 and BRCA 2 mutations as well as mutations in the ATM, CDH1, CHEK2, TP53, PTEN, PALB2, and STK11 genes.

**Figure 1:**
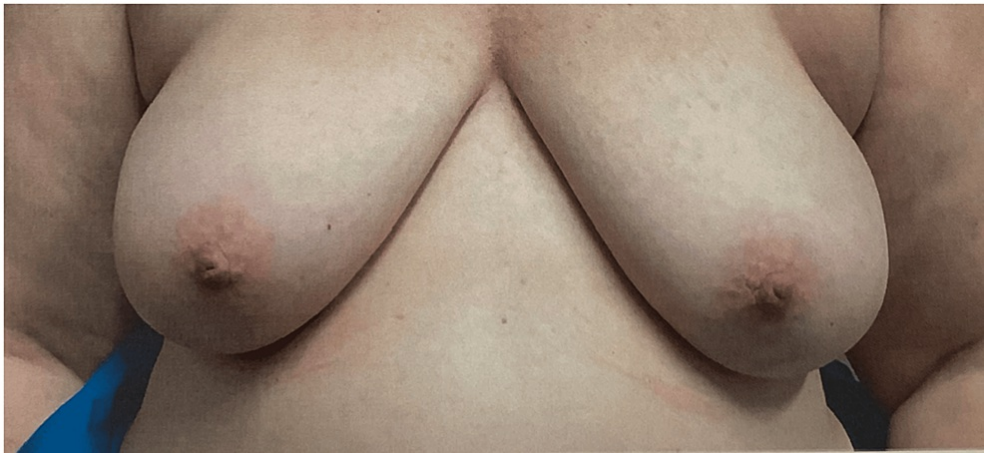
Pre-operative Image of Patient’s Breasts Prior to Bilateral Mastectomy with Immediate Reconstruction Pre-operative photograph of the patient’s breasts is shown, documenting the condition before undergoing bilateral mastectomy with immediate reconstruction. The image was taken to assess the breast tissue and to aid in surgical planning. There was no palpable cervical, supraclavicular, or axillary lymphadenopathy, indicating no detectable enlargement of the lymph nodes in these regions upon clinical examination.

Physical exam of the breasts revealed symmetry, no skin changes, no nipple discharge, and no suspicious dominant palpable masses. There was also no palpable cervical, supraclavicular, or axillary lymphadenopathy (Figure 1). A mammogram of the left breast revealed a 2.9 x 1.8 x 1.4 cm group of pleomorphic segmental calcifications in the anterior lower outer quadrant along with a posterior 8 mm oval circumscribed mass 3 o’clock position which was later discovered to be a benign intramammary lymph node on ultrasound. Ultrasound of the left breast revealed a 9 x 7 x 3 mm hypoechoic mass at the 3 o’clock position as well as a 5 x 5 x 4 mm FN incidentally found hypoechoic mass at the 3 o’clock position. Ultrasound of the right breast revealed a 4 mm group of faint calcifications in the central breast at middle depth. This right breast mass was determined to be non-cancerous through MRI-guided biopsy.

A subsequent biopsy of the mass in the left breast revealed DCIS, poorly differentiated - grade 3 with associated microcalcifications. Per the patient’s request, a bilateral non-nipple sparing mastectomy was performed instead of a left lumpectomy. Ultimately, the patient underwent a left breast mastectomy with removal of two left axillary sentinel lymph nodes (Table 1) and a right breast prophylactic mastectomy. The mastectomies were done through breast reduction type of incisions with de-epithelialized flaps inferiorly. At the time of the operation, the tumor was behind the areola and measured 2.9 cm. After the removal of breast tissue, the patient underwent bilateral reconstruction alongside the mastectomy with tissue expander, which incorporated a non-displaced-bearing breast reduction-type incision and prosthetic-based reconstruction.

**Table 1:** Characteristics of Left Axillary Sentinel Lymph Nodes Characteristics of the left axillary sentinel lymph nodes excised during surgery are detailed. Lymph node #1 measures 1.2 x 1.0 x 1.0 cm and appears as a yellow fatty lymph node with attached yellow adipose tissue. Microscopic examination revealed findings noted as All/2, with no granulomas (G). Lymph node #2 measures 3.8 x 2.6 x 1.5 cm, presenting as a red-brown lymph node with attached yellow adipose tissue. Microscopic examination for this node revealed findings noted as All/5, also with no granulomas.

Specimen identifier	Pathology finding(s)
Left axillary sentinel lymph node # 1	1.2 x 1 x 1 cm yellow fatty lymph node with attached yellow adipose tissue All/2, no G
Left axillary sentinel lymph node # 2	3.8 x 2.6 x 1.5 cm red-brown lymph node with attached yellow adipose tissue All/5, no G

**Figure 2:**
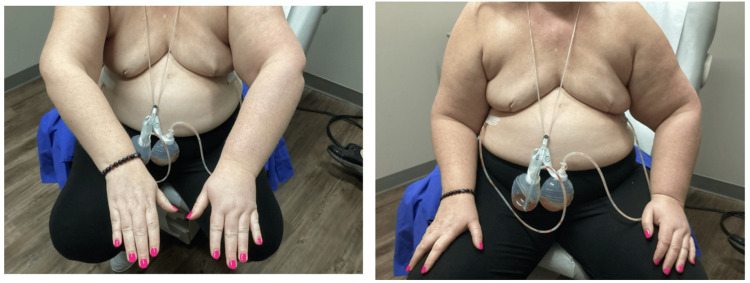
Post-operative Images of the Patient After Bilateral Mastectomy Post-operative photographs of the patient following bilateral mastectomy are shown. The left arm appears pink and warm yet exhibits 2+ non-pitting edema in the left hand and forearm. The patient reported swelling and pain in the arm, with left-sided edema extending from the clavicle to the fingers.

**Figure 3:**
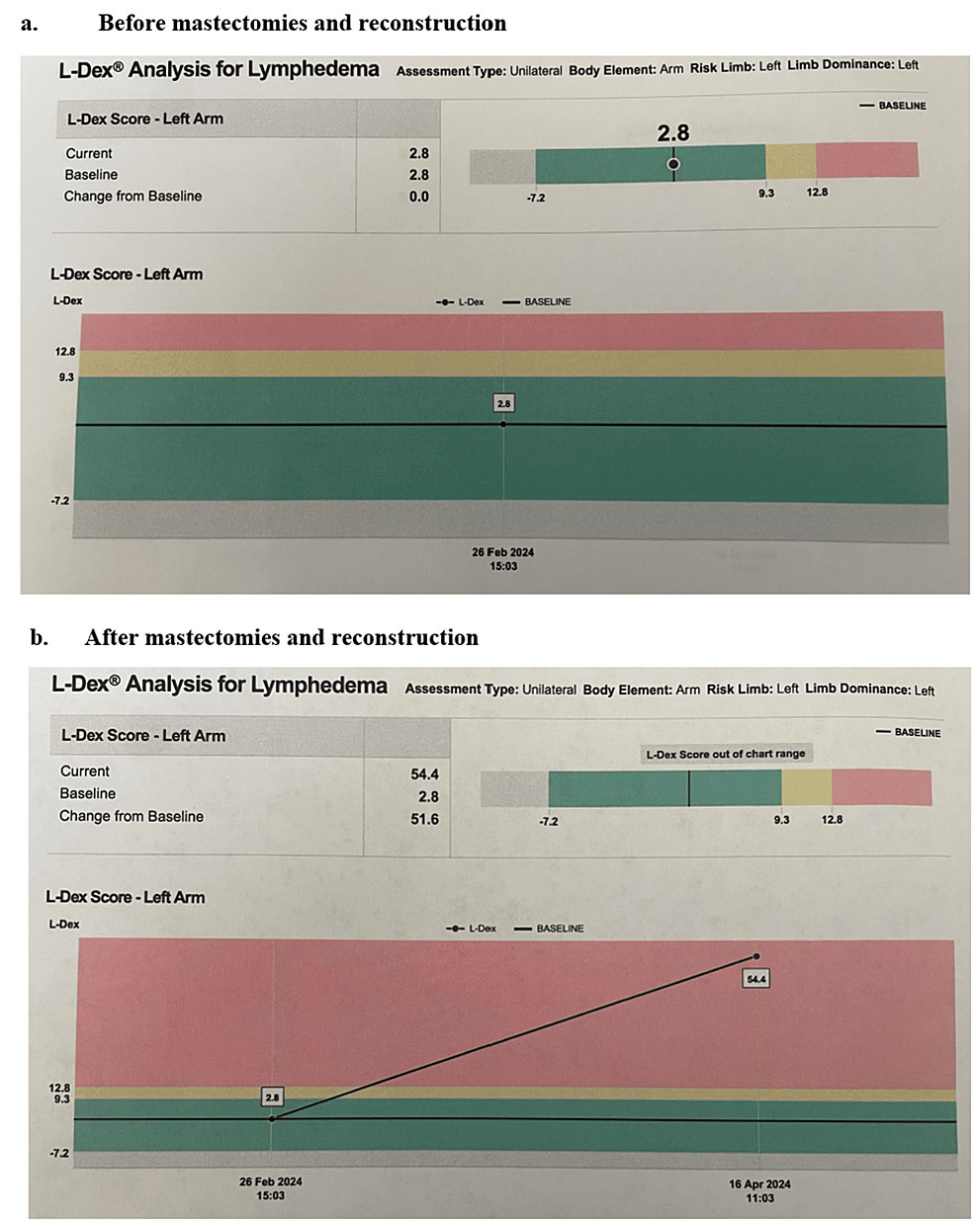
SOZO Measurements Pre- and Post-operation SOZO measurements of the patient are presented in two parts: (a) pre-operation and (b) post-operation. The SOZO device uses bioimpedance spectroscopy to assess fluid status and body composition. Pre-operative measurement (a) shows a reading of 2.8, indicating normal fluid levels. Post-operative measurement (b) shows a significant increase to 54.4, reflecting a very large increase in extracellular fluid.

Two weeks after the procedure, the patient presented for a follow-up appointment for arm swelling and pain with left-sided edema extending from the clavicle to the fingers with the left radial pulse being +2 and regular. On the physical exam, the left arm was pink and warm, but the patient had 2+ non-pitting edema in her left hand and left forearm (Figure 2). SOZO (brand name for a device developed by ImpediMed) measurements, which are meant to quantify the risk of developing lymphedema through non-invasive bioimpedance spectroscopy in order to detect lymphedema at the preclinical stage and allow for early intervention with decompressive therapy and compression garments to reduce the progression of lymphedema by 95% [5], were performed before and after the mastectomies (Figure 3). A Doppler ultrasound was then ordered and revealed acute deep venous thrombosis in the left axillary and brachial veins extending from the proximal to the distal aspects, acute superficial venous thrombosis in the left basilic vein of the upper arm, and acute non-occlusive thrombus in the median cubital vein. The patient was treated in time with Heparin and Eliquis and reported for a later follow-up appointment without any complaints of pain or swelling in the left arm. 

## Discussion

Unlike other breast malignancies, DCIS is an indolent, non-invasive form of breast cancer with very high overall survival rates (98% 25-year survival [6]). Like other breast malignancies, DCIS can display ER, progesterone (PR), and/or human epidermal growth factor receptor 2 (HER2) receptor activity. It has been noted that 80 to 95% of DCIS occurrences express ER receptors [7] and 60% express PR receptors [8]. Therefore, after the surgical removal of this pre-malignancy, patients can be effectively managed by endocrine therapy with drugs like tamoxifen, or one of the aromatase inhibitors such as anastrozole, exemestane, and letrozole. All in all, it is highly unlikely that the mammographically detected DCIS in this patient resulted in the upper extremity DVT. In DCIS, the malignant cells are confined to the basement membrane of the breast ducts [9] and have not spread lymphatically or hematogenously. 

From a hematology standpoint, coagulopathy is much more likely to have caused the DVT in this patient. Upper extremity DVT accounts for only 6% of total occurrences [10], and the instances of them occurring alongside DCIS are minuscule. There is very little research documenting such cases. What happened to this patient was very unique. Hereditary causes of hypercoagulability that predispose patients to venous thrombosis such as factor V Leiden, protein C deficiency, or antithrombin III deficiency [11] are most likely what contributed to the thrombus formation in this patient. An autoimmune source of DVT could have been antiphospholipid syndrome where antibodies create complexes with anticoagulants such as antithrombin III, protein C, and protein S, thereby inactivating them and increasing the risk for thrombosis and embolism [12]. From a lymphatic standpoint, it was highly unlikely that the swelling and pain experienced by this patient were the result of removing only two axillary lymph nodes. Post-operative lymphedema is a chronic complication that develops over the course of months to years, not weeks, as fluid accumulates in the interstitial tissue. Therefore, lymphedema develops progressively with a subclinical phase that can last for years before any physical manifestations appear [13]. It is in her best interest to undergo a CBC, BMP, liver function tests, blood factor assays, a peripheral blood smear, PT/INR, and aPTT as soon as possible to uncover the root cause of her upper extremity DVT [14]. 

## Conclusions

Going forward, continuity of care will be very important for this patient. She will need to be continuously monitored by providers specializing in hematology and oncology. It is in her best interest to undergo a CBC, BMP, liver function tests, blood factor assays, a peripheral blood smear, PT/INR, and aPTT to uncover the root cause of her upper extremity DVT. The patient should also be regularly screened with SOZO measurements in order to prophylactically treat lymphedema should it ever appear. Early detection of issues with lymphatic drainage and reabsorption are reversible and would be of the most benefit to the patient. Due to undergoing bilateral mastectomies, she will no longer require yearly mammograms as she no longer has native breast tissue. However, constant physical examinations of the breasts will need to be done alongside follow-ups with providers in order to proactively address any issue in the future and avoid further complications. Although upper extremity DVT may be rare in the context of breast cancer, healthcare providers should remain vigilant and consider it as a potential concern. It is essential to acknowledge that upper extremity DVT can occur post-mastectomies. There is certainly a need for preventive guidance in future breast cancer patient management.
